# Spatially Enhanced Differential RNA Methylation Analysis from Affinity-Based Sequencing Data with Hidden Markov Model

**DOI:** 10.1155/2015/852070

**Published:** 2015-08-02

**Authors:** Yu-Chen Zhang, Shao-Wu Zhang, Lian Liu, Hui Liu, Lin Zhang, Xiaodong Cui, Yufei Huang, Jia Meng

**Affiliations:** ^1^Key Laboratory of Information Fusion Technology of Ministry of Education, School of Automation, Northwestern Polytechnical University, Xi'an 710072, China; ^2^School of Information and Electrical Engineering, China University of Mining and Technology, Xuzhou 221116, China; ^3^Department of Electrical and Computer Engineering, University of Texas at San Antonio, San Antonio, TX 78249, USA; ^4^XJTLU-WTNC Research Institute, Department of Biological Sciences, Xi'an Jiaotong-Liverpool University, Suzhou 215123, China

## Abstract

With the development of new sequencing technology, the entire N6-methyl-adenosine (m^6^A) RNA methylome can now be unbiased profiled with methylated RNA immune-precipitation sequencing technique (MeRIP-Seq), making it possible to detect differential methylation states of RNA between two conditions, for example, between normal and cancerous tissue. However, as an affinity-based method, MeRIP-Seq has yet provided base-pair resolution; that is, a single methylation site determined from MeRIP-Seq data can in practice contain multiple RNA methylation residuals, some of which can be regulated by different enzymes and thus differentially methylated between two conditions. Since existing peak-based methods could not effectively differentiate multiple methylation residuals located within a single methylation site, we propose a hidden Markov model (HMM) based approach to address this issue. Specifically, the detected RNA methylation site is further divided into multiple adjacent small bins and then scanned with higher resolution using a hidden Markov model to model the dependency between spatially adjacent bins for improved accuracy. We tested the proposed algorithm on both simulated data and real data. Result suggests that the proposed algorithm clearly outperforms existing peak-based approach on simulated systems and detects differential methylation regions with higher statistical significance on real dataset.

## 1. Introduction

Although the presence of posttranscriptional biochemical modifications to RNA has been established in 1960s [1], due to historical limitations, RNA epigenetics is largely uncharted territory until recently [2–4]. In 2012, a powerful sequencing protocol methylated RNA immune-precipitation sequencing (MeRIP-Seq or m^6^A-Seq) was developed [5, 6], in which the fragmented mRNA fragments with N6-methyl-adnosine (m^6^A) are pulled down with anti-m^6^A antibody and then purified and passed to subsequent sequencing to generate the so-called “IP sample” for profiling the transcriptome-wide RNA m^6^A methylome. Very often, a paired “input sample” is generated as well using all the RNA for measuring the entire transcriptome background (please refer to [7] for a more comprehensive protocol of this approach). This technique facilitates a number of research findings recently which includes the following: the role of RNA methylation in controlling the circadian clock [8], addiction [9], and stem cell [10], and [2, 3, 5, 6, 8–16]. It also enabled the construction of mammalian RNA methylation database [17] and systems biology approaches for decomposing the RNA methylome to unveil the latent enzymatic regulators of epitranscriptome [18]. Software tools for RNA methylation site detection [19, 20] and for differential RNA methylation analysis [21] from MeRIP-Seq data are now available in a rather user friendly manner. Nevertheless, as a newly arising technique, MeRIP-Seq still poses computational challenges that call for novel and sophisticated approaches.

**Figure 1:**
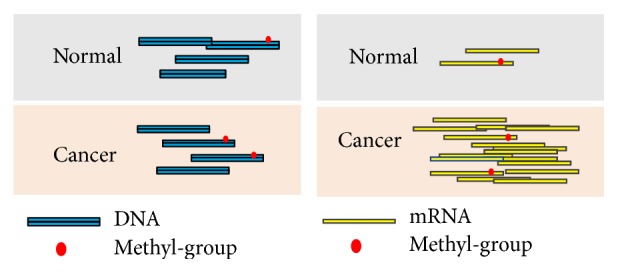
Comparison of the differential methylation analysis in DNA and RNA. The first column shows the DNA related differential analysis in ChIP-Seq or MeDIP-Seq, where the total DNA is often considered the same under two experimental conditions, so the differential analysis can be performed by directly comparing the absolute amount of methylated RNAs in the two IP samples. In contrast, for RNA (the second column), the background is total RNA, which can vary significantly under different conditions, and therefore, the absolute amount of methylated RNA for a specific site does not necessarily correlate with the degree of methylation. For the example shown in the above figure, while amount of methylated RNA increases under the cancer condition, the relative amount (percentage of methylated RNA) decreases, indicating a hypomethylation at RNA level. As a result, the differential analysis of RNA methylome in MeRIP-Seq should be performed by comparing the percentages of methylated RNA to reflect the influence of methylation enzymatic regulation.

Differential methylation analysis is of crucial importance for epigenetics research. Differentially methylated regions (DMRs), that is, regions that exhibit different methylation levels between two experimental conditions, for example, normal and cancerous, can be as small as a single base or as large as an entire gene locus, depending on the biological question of interest and the bioinformatics methods used for their identification [22]. Differential methylation analysis from MeRIP-Seq seeks to identify the differences in RNA methylome in a case-control study (e.g., cancerous and normal), which usually involves at least four high-throughput sequencing (HTS) samples, including the IP and input samples under both the case and control conditions. For affinity-based methods developed for DNA epigenetics (such as MeDIP-Seq and ChIP-Seq), since the absolute amount of DNA is most likely to stay unchanged between two conditions, the percentage of modified DNA molecule is linearly correlated with the absolute amount; thus the difference in methylation is consistent when measured in relative (percentage) and absolute amount. However, in MeRIP-Seq, due to the change in transcriptional expression level between two conditions, it is possible that while the absolute amount of methylated RNA increases, the relative amount (percentage of methylated RNA) decreases as shown in Figure 1. From computational perspective, the differential methylation analysis of RNA is quite different from that of DNA, and DNA differential methylation approaches [23], such as MOABS [24] and DMAP [25], may not be directly applicable to RNA. Until now, methods aiming at the differential analysis of MeRIP-Seq data do not extensively appear in literature. exomePeak [19, 21] is dedicatedly developed for differential RNA methylation analysis from MeRIP-Seq data. The detection of DMRs is based on rhtest [26], which is an extended version of hypergeometric test, computing the statistical significance of the difference in the percentages of methylated fragments between the two conditions, which directly indicates the difference in enzymatic regulation. Before the detection of DMRs, peaks (methylated regions) are called firstly from the transcriptome by comparing the IP with input sample by relative enrichment [7, 19, 27]. Only with the detected methylation sites can we effectively estimate the methylation level.

**Figure 2:**
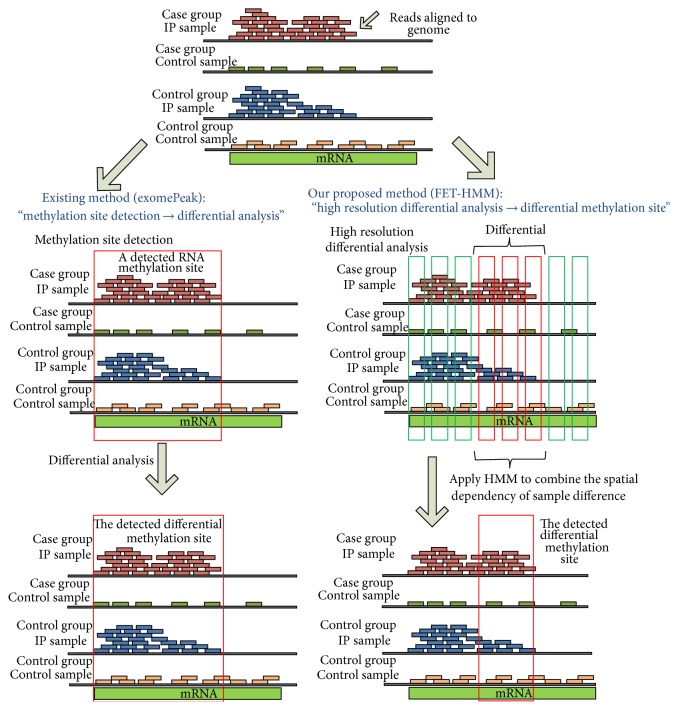
Comparison of differential methylation analysis methods. This figure shows the difference between existing peak-based differential analysis method and the proposed method. Started from aligned reads, the left part of this figure shows how exomePeak conducts differential analysis. It firstly identifies a single methylation site and then decides whether the methylation site as a whole is differentially methylated or not. However, the newly proposed method will split the testing region into multiple adjacent small bins and then will integrate their dependency with HMM for more accurate identification of differential methylation site. In the above example, the RNA methylation site detected using exomePeak method may consist of two methylation residuals, and only the one on the right side is differentially methylated in this case-control study. The proposed FET-HMM method is likely to work better than peak-based exomePeak method under this scenario.

Affinity-based approaches cannot provide single-base resolution. Since multiple RNA methylation residuals may locate in proximity and cannot be effectively differentiated with peak calling procedure, they can appear as a single broad methylation site in the peak calling result from MACS [27] or exomePeak [19]. In many cases, this discrepancy can be trivial and does not significantly affect relevant study; however, it can be disastrous in differential methylation analysis, because multiple RNA methylation residuals can be regulated by different enzyme complexes and thus may be differentially methylated. Failing to identify the precise location of each methylation residual can lead to large bias in the estimation of its methylation level and in the comparison to a different condition. Currently, all existing methods for RNA differential methylation from MeRIP-Seq data are peak-based. In this paper, based on the rhtest method developed in exomePeak package [21], we proposed FET-HMM, a novel strategy for spatially enhanced differential RNA methylation analysis using hidden Markov model (HMM). When applying to the RNA methylation site detected from a peak calling algorithm, FET-HMM breaks a single site into multiple adjacent small bins and evaluates whether a specific bin is differentially methylated or not between two experimental conditions with spatial dependency incorporated by HMM.  Figure 2 shows the comparison between existing and our methods.

HMM is a statistical model that integrates multiple random processes and has been widely used in DNA-templated epigenetic analysis and in RNA methylation sites detection (or peak calling) [28–30], but so far it has not been applied for RNA differential methylation analysis. We applied the newly developed approach FET-HMM on both simulated and real datasets. The results on simulated data showed that FET-HMM can effectively improve the performance of rhtest in terms of the area under the curve (AUC) when detecting differential methylation sites. When applied to human MeRIP-Seq datasets, FET-HMM method returns more biological meaningful results than exomePeak method. The FET-HMM algorithm has been implemented in an open source R package for differential methylation analysis from MeRIP-Seq data and is freely available from GitHub. The method is detailed in the following section.

## 2. Methods

In this section, we firstly review the usage of rhtest, a modified version of Fisher's exact test (FET), for differential RNA methylation analysis and then introduce spatially enhanced approach FET-HMM.

### 2.1. Peak-Based Differential RNA Methylation Analysis with Rhtest

To conduct differential RNA methylation analysis in a case-control study, we should get four samples, that is, the IP and input samples from both groups. Consider that there are a number of RNA methylation sites detected with peak calling approaches [19, 20, 27] from MeRIP-Seq. Then we can assume that the number of reads within the *g*th RNA methylation sites follows the Poisson distribution, with

(1) $$\begin{array}{c} X_{0,g}\sim \text{Poisson}\left(N_{0}\lambda_{0,g}\right),\\ X_{1,g}\sim \text{Poisson}\left(N_{1}\lambda_{1,g}\right),\\ Y_{0,g}\sim \text{Poisson}\left(M_{0}{\overset{-}{\lambda }}_{0,g}\right),\\ Y_{1,g}\sim \text{Poisson}\left(M_{1}{\overset{-}{\lambda }}_{1,g}\right), \end{array}$$

where *X*_0,*g*_ and *X*_1,*g*_ are the reads counts of the input samples for untreated and treated condition and consistently, *Y*_0,*g*_ and *Y*_1,*g*_ are the reads counts of the IP samples for untreated and treated samples. Here, *g* = 1,2,…, *G* indicates the *g*th RNA methylation site. (*N*_0_, *N*_1_, *M*_0_, *M*_1_) are the size (or the sequencing depth) of library, respectively; and the parameters $\left(\lambda_{0,g},\lambda_{1,g},{\overset{-}{\lambda }}_{0,g},{\overset{-}{\lambda }}_{1,g}\right)$ are the normalized Poisson means in a standard library, indicating the expectation of the reads counts within a bin. Following the formulation from previous study [26], we assume that ${\overset{-}{\lambda }}_{0,g}$ and ${\overset{-}{\lambda }}_{1,g}$ satisfy the following relationship with ${\overset{-}{\lambda }}_{0,g}=\lambda_{0,g}\eta_{0,g}/f_{0}$ and ${\overset{-}{\lambda }}_{1,g}=\lambda_{1,g}\eta_{1,g}/f_{1}$, where *f*_0_ and *f*_1_ indicate the percentage of the expressed RNA fragments that are modified in the untreated and treated samples, respectively. *η*_0,*g*_ and *η*_1,*g*_ indicate the percentage of RNA fragments mapped inside the RNA methylation site that carry the methylation mark. We would like to test whether *η*_1,*g*_ = *η*_0,*g*_. According to the properties of the Poisson distributions [31, 32], given *X*_0,*g*_ + *Y*_0,*g*_ = *t*_0,*g*_, *X*_1,*g*_ + *Y*_1,*g*_ = *t*_1,*g*_, we should have *X*_0,*g*_ ~ Binomial(*p*_0,*g*_, *t*_0,*g*_) and *X*_1,*g*_ ~ Binomial(*p*_1,*g*_, *t*_1,*g*_), where *p*_1,*g*_ = *N*_1_ *f*_1_/(*N*_1_ *f*_1_ + *M*_1_ *η*_1,*g*_) and *p*_0,*g*_ = *N*_0_ *f*_0_/(*N*_0_ *f*_0_ + *M*_0_ *η*_0,*g*_). For different experimental conditions, if we assume that the total amount of modifications remains the same, only its distribution may change, then we can have *f*_0_ = *f*_1_ = *f*. We also notice that if *N*_1_ *M*_0_ = *N*_0_ *M*_1_, then *η*_1,*g*_ = *η*_0,*g*_⇔*p*_1,*g*_ = *p*_0,*g*_, and testing whether the two Binomial distributions have the same successful rate is equivalent to the classical problem of testing the independence in a 2 × 2 contingency table. In order to establish *N*_1_ *M*_0_ = *N*_0_ *M*_1_, only one of the 4 samples needs to be rescaled. When *N*_1_ *M*_0_ = *N*_0_ *M*_1_ is achieved after rescaling, under the null hypothesis *p*_1,*g*_ = *p*_0,*g*_, *X*_0,*n*_ follows a hypergeometric distribution as in (2), and we may use Fisher's exact test [33–36] with two tails to evaluate its significance. Consider

(2) $$\begin{array}{c} p\left(X_{0,g}=k\right)\sim \text{Hyper}\left(X_{0,g}\;|\;K,n,N\right)=\frac{\left(\begin{array}{c} K\\ k \end{array}\right)\left(\begin{array}{c} N-K\\ n-k \end{array}\right)}{\left(\begin{array}{c} N\\ n \end{array}\right)}, \end{array}$$

where *N* = *t*_0,*g*_ + *t*_1,*g*_ = *x*_0,*g*_ + *x*_1,*g*_ + *y*_0,*g*_ + *y*_1,*g*_, *n* = *t*_0,*g*_ = *x*_0,*g*_ + *y*_0,*g*_, and *K* = *x*_0,*g*_ + *x*_1,*g*_. The smaller the *p* value is, the more likely the *g*th RNA methylation site is differentially methylated between two conditions.

### 2.2. Spatially Enhanced Differential RNA Methylation Analysis with FET-HMM

The method developed in the previous section could not effectively discriminate multiple RNA methylation residuals located within a single RNA methylation site (as shown in Figure 1). We seek to enhance the spatial resolution with hidden Markov model. Similar to various formulation, for a particular RNA methylation site, we firstly divided it into *N* mutually connected bins of length *L*. Then we can still assume that the number of reads within the *n*th bin follows the Poisson distribution, with

(3) $$\begin{array}{c} X_{0,n}\sim \text{Poisson}\left(N_{0}\lambda_{0,n}\right),\\ X_{1,n}\sim \text{Poisson}\left(N_{1}\lambda_{1,n}\right),\\ Y_{0,n}\sim \text{Poisson}\left(M_{0}{\overset{-}{\lambda }}_{0,n}\right),\\ Y_{1,n}\sim \text{Poisson}\left(M_{1}{\overset{-}{\lambda }}_{1,n}\right), \end{array}$$

where *X*_0,*n*_ and *X*_1,*n*_ are the reads counts of the input samples for untreated and treated condition and consistently, *Y*_0,*n*_ and *Y*_1,*n*_ are the reads counts of the IP samples for untreated and treated samples. Here, *n* = 1,2,…, *N* indicates the *n*th bin. The parameters $\left(\lambda_{0,n},\lambda_{1,n},{\overset{-}{\lambda }}_{0,n},{\overset{-}{\lambda }}_{1,n}\right)$ are the normalized Poisson means in a standard library, indicating the expectation of the reads counts within a bin. Following the formulation from previous study [26], we assume that ${\overset{-}{\lambda }}_{0,n}$ and ${\overset{-}{\lambda }}_{1,n}$ satisfy the following relationship with ${\overset{-}{\lambda }}_{0,n}=\lambda_{0,n}\eta_{0,n}/f_{0}$ and ${\overset{-}{\lambda }}_{1,n}=\lambda_{1,n}\eta_{1,n}/f_{1}$, where *f*_0_ and *f*_1_ indicate the percentage of the expressed RNA fragments that are modified in the untreated and treated samples, respectively. *η*_0,*n*_ and *η*_1,*n*_ indicate the percentage of RNA fragments mapped inside the bin that carry the methylation mark. We can easily test whether *η*_1,*n*_ = *η*_0,*n*_ (whether differential methylation is observed) for a specific bin; however, we should not neglect the dependencies between the reads counts of adjacent bins within an RNA methylation site; that is, if differential methylation is observed on a specific bin, it is likely that differential methylation can also be observed on bins adjacent to it and vice versa. The dependency can be effectively incorporated with an HMM formulation, and we thus developed a new strategy for the identification of differential methylation regions (DMRs) with improved spatial resolution.

To begin with, with respect to *n*th bin, the hidden true states of differential methylation are denoted as *S* = {*s*_1_, *s*_2_,…, *s*_*N*_}, where *s*_*n*_ ∈ {0,1} with 1 representing differential methylation state (DMS) and 0 otherwise. Considering that a differential methylation region may span multiple adjacent bins, we assume that the true hidden DMS *S* follows a first order Markov chain, whose transition matrix *A* contains entries defined as

(4) $$\begin{array}{c} A_{ij}=P\left(s_{n+1}=j\;|\;s_{n}=i\right),\;i,j\in \left\{0,1\right\}, \end{array}$$

where *A*_*ij*_ denotes the probability for the hidden variable switching from DMS *i* at the *n*th bin to the DMS *j* at the (*n* + 1)th bin. In addition, the initial probability *p*(*S*_1_ = 0) = *u* and *p*(*S*_1_ = 1) = 1 − *u*, which can be denoted as *π* = (*u*, 1 − *u*). Next, the result of rhtest [21, 26] was used as the observed variable of the HMM. However, the information acquired from rhtest is a statistical significance of differential methylation in terms of *p* values and FDRs (False Discovery Rates). We seek to enhance the differential methylation results by incorporating spatial dependency. Specifically, 3 different strategies are developed for this purpose with their own advantages and disadvantages, which are detailed in the following.

### 2.3. FHB Strategy: Combine Fisher's Exact Test and HMM with Binary Observation

In FHB strategy, we use the binary decisions received from FET as the observation of hidden Markov model. The model essentially evaluates how likely a true differential methylation state can be detected by FET, or if FET reports a DMS with a significance level, how likely it is true after incorporating spatial dependency. We assume that a state can be correctly observed with probability *p*; and a mistake happens with probability (1 − *p*). Since the observation from FET is considered as binary, a cut-off threshold should be used to switch the FDR (False Discovery Rate) value to generate the “observed” set of observed variable *O* = (*o*_1_, *o*_2_,…, *o*_*n*_) with *o*_*n*_ ∈ {0,1}. Then according to the standard HMM definition, these probabilities consist of an emission matrix *B*, whose entries are defined as

(5) Bij=Pon=j ∣ sn=i=p,i,j∈0,1,  i=j,1−p,i,j∈0,1,  i≠j.

**Figure 3:**
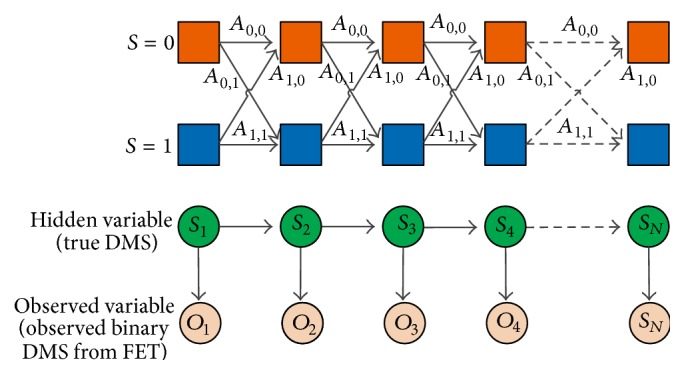
Hidden Markov model. In FHB strategy, the “observation” is a binary status reported from FET, and the emission probability is Bernoulli distribution.

The detailed structure of HMM is shown in Figure 3.

Finally, we applied the widely used Baum-Welch algorithm [37–39] to estimate the unknown parameters of the HMM. Baum-Welch algorithm applies the well-known Expectation and MSaximization (EM) strategy to conduct the process of estimation. The implementation steps of Baum-Welch algorithm are as follows.

*The Proposed Algorithm*

*(1) Initialization.* Given the initial value of *A*_*ij*_, *π*_*i*_, and *B*_*ij*_ randomly according to the conditions of probability, we hence get the initial model parameters *λ* ^(0)^ = (*π* ^(0)^, *A* ^(0)^, *B* ^(0)^).

*(2) EM Steps*

*E Step.* Let *γ*_*n*_(*i*) denote the probability of the hidden DMS being at *i* at the *n*th bin, and let *ξ*_*n*_(*i*, *j*) denote the probability of the hidden DMS being at *i* at the *n*th bin and the DMS being at *j* at the (*n* + 1)th bin. Also, we denote *ts*_*ik*_, *k* ∈ {0,1}, to represent the times of the transition from DMS *i* to any DMS *k* and *ts*_*ij*_ to represent the times of the transition from DMS *i* to the DMS*j*. *γ*_*n*_(*i*) and *ξ*_*n*_(*i*, *j*) can be computed through (6) and (7), and the expectation of *ts*_*ik*_ and *ts*_*ij*_ can be calculated by (8) and (9). *λ* ^(*m*)^ = (*π* ^(*m*)^, *A* ^(*m*)^, *B* ^(*m*)^) represents the parameters of HMM after the *m*th iteration. Consider

(6) $$\begin{array}{c} \gamma_{n}\left(i\right)=P\left(s_{n}=i\;|\;O,\lambda^{\left(m\right)}\right)=\frac{P\left(s_{n}=i,\;\;O\;|\;\lambda^{\left(m\right)}\right)}{P\left(O\;|\;\lambda^{\left(m\right)}\right)}, \end{array}$$

(7) ξni,j=Psn=i,  sn+1=j ∣ O,λm=Psn=i,  sn+1=j,  O ∣ λmPO ∣ λm,

(8) $$\begin{array}{c} E\left[t_{s_{ik}}\right]=\overset{N-1}{\underset{n=1}{\sum }}\gamma_{n}\left(i\right), \end{array}$$

(9) $$\begin{array}{c} E\left[t_{s_{ij}}\right]=\overset{N-1}{\underset{n=1}{\sum }}\xi_{n}\left(i,j\right). \end{array}$$

*M Step.* After using (10), (11), and (12) to estimate *π*_*i*_, *A*_*ij*_, and *B*_*ij*_, we get *λ* ^(*m*+1)^. One has

(10) $$\begin{array}{c} {\pi_{i}}^{\left(m+1\right)}=\gamma_{1}\left(i\right), \end{array}$$

(11) $$\begin{array}{c} {a_{ij}}^{\left(m+1\right)}=\frac{E\left[t_{s_{ij}}\right]}{E\left[t_{s_{ik}}\right]}=\frac{\sum_{n=1}^{N-1}\xi_{n}\left(i,j\right)}{\sum_{n=1}^{N-1}\gamma_{n}\left(i\right)}, \end{array}$$

(12) $$\begin{array}{c} {b_{i}}^{\left(m+1\right)}\left(k\right)=\frac{\sum_{n=1}^{N}\gamma_{n}\left(i\right)I_{\left\{o_{n}=k\right\}}}{\sum_{n=1}^{N}\gamma_{n}\left(i\right)}. \end{array}$$

In (12),

(13) I{on=k}=1on=k0on≠k

is the indicative function.

*(3) Loop.* Repeat the EM steps until the convergence of *A*_*ij*_, *π*_*i*_, and *B*_*ij*_. After the procedures above, optimal model parameter *λ* ^(op)^ could be obtained. Let *u*_*nk*_ = 1 if we are absolutely sure *s*_*n*_ = *k* and *u*_*nk*_ = 0 otherwise. What we focused on is the final expectation of *u*_*nk*_, *k* ∈ {0,1}, which can be calculated as

(14) $$\begin{array}{c} E\left[u_{nk}\;|\;O,\lambda^{\left(\text{op}\right)}\right]=P\left(s_{n}=k\;|\;O,\lambda^{\left(\text{op}\right)}\right). \end{array}$$

Then we could obtain the posterior probability of a bin being at a specific state, and the performance of FET-HMM can be compared with that of exomePeak on simulated dataset when the true state is available.

### 2.4. FHC Strategy: Combine Fisher's Exact Test and HMM with Continuous Observation

In FHB strategy, we adopt a switching cut-off threshold to convert the statistical significance (*p* value from differential analysis with rhtest) into binary states as the observation of HMM. This strategy has two limitations. Firstly, we could hardly find the most reasonable threshold for a dataset, and different threshold can lead to different results. Secondly, some information gets lost in the conversion from *p* value to binary states; for example, both *p* values 0.01 and 0.001 are converted as DMS state 1 after a binary conversion with significance level 0.05; however, the former is less confident. In addition, Bernoulli distribution may not be the most suitable distribution for the emission probability of observed variable. Therefore, a strategy seeking to directly smooth the continuous statistical significance without binary conversion may be superior. For this purpose, we use the *p* values from FET to approximate the likelihood of a bin with DMS state 0 and (1 − *p*  value) for its likelihood with DMS state 1. The *p* values generated from FET can be used to estimate the emission probability of HMM directly and then passed to HMM for smoothing purposes. It should be denoted as

(15) $$\begin{array}{c} B_{\text{II}}=\left[\begin{array}{cc} p\;\;{\text{value}}_{1}\; & 1-p\;\;{\text{value}}_{1}\\ p\;\;{\text{value}}_{2}\; & 1-p\;\;{\text{value}}_{2}\\ \vdots & \vdots \\ p\;\;{\text{value}}_{N} & 1-p\;\;{\text{value}}_{N} \end{array}\right]. \end{array}$$

After getting the matrix *B*_II_ of size *N* by 2 constructed from FET *p* values, the Baum-Welch algorithm introduced in FHB can be applied to spatially enhance the local result, with formula (12) omitted because matrix *B*_II_ does not need to be reestimated every iteration. Please note that using *p* values to approximate directly the probability matrix *B*_II_ helps to avoid the binary conversion and information loss, and we will show in the Result section that this trick indeed improves the performance of algorithm.

### 2.5. FastFH Strategy: A High-Efficiency Strategy for Applying FET-HMM on Big Omics Data

When the proposed method is used in real MeRIP-Seq dataset, two problems would emerge. What comes first was some reads would be mapped into very short genes; thus the number of the bins would be quite small. In other words, the length of some Markov chains would be too short for accurate estimation of parameters and finally affects the results of DMRs detection. In addition, computational time was another important factor that we should take into consideration. Take the human hg19 data we were going to test as an example. If there were more than 30000 detected RNA methylation sites in total, the Baum-Welch algorithm would be performed more than 30000 times and the execution time might be too long. In order to solve these two limitations, we could combine the two strategies together. Firstly, the threshold used in FHB was used here again to switch the FDR into binary DMS. Then we could estimate transition matrix *A*_III_ directly from this DMS information as shown in

**Figure 4:**
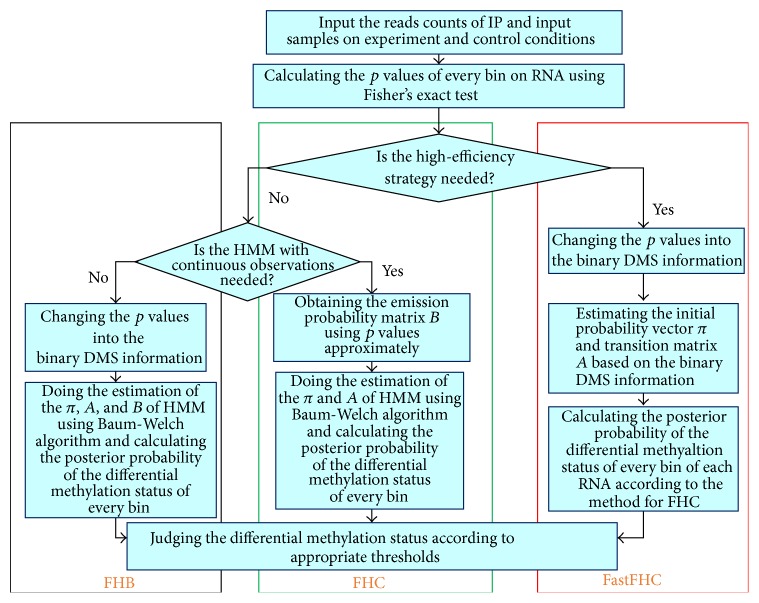
Comparison of different strategies. FHB strategy is the most naïve and straightforward; FHC is the most time consuming and performs better than FHB but is less robust. With FastFHC, the algorithm can now be applied to genome scale dataset in a timely and robust manner.

(16) πIII=1−∑i=1NDMSiN,∑i=1NDMSiN,AIII=PSn+1=0 ∣ Sn=0PSn+1=1 ∣ Sn=0PSn+1=0 ∣ Sn=1PSn+1=1 ∣ Sn=1,

where *P*(*S*_*n*+1_∣*S*_*n*_) denotes the conditional probability for the transition from *S*_*n*_ to *S*_*n*+1_, which can be conveniently estimated by scanning all the states of differential methylation *S* = {*s*_1_, *s*_2_,…, *s*_*N*_} on all RNA methylation sites. For every single gene, the emission probability *B*_III_ has the same form as *B*_II_ in FHC strategy. By doing this, the *A*_III_ matrix can be estimated in a single step instead of an iterative manner so as to save computation load. This result should be also more robust on short RNA methylation sites with less number of bins than previous strategy. Secondly, we chose the Estep in FHB strategy to compute the final expectation defined in formula (14) for every single bin on every RNA methylation sites of real RNA epigenetics data. FastFHC strategy applied Estep after estimating transition matrix and initial probability for all genes. *π*_III_ and *A*_III_ are considered the same on different RNA methylation sites and are estimated like FHB with binary converted observation. Although some information can be lost in the conversion step, since tens of thousands of RNA methylation sites are pooled together for estimation of *π*_III_ and *A*_III_, it should be still relatively accurate. The 3 strategies are summarized in Figure 4.

## 3. Result

### 3.1. Test on Simulated Data

For MeRIP-Seq, as the ground truth is not available for the differential RNA methylation status in real data, the performance of our proposed method (FHB and FHC strategy) was first validated on simulated datasets. Specifically, the reads counts for the IP and input samples under two experimental conditions were generated from model assumptions, respectively. In every set of data, 100 RNA methylation sites are generated, each with 1000 adjacent bins. The sequencing depths were all set 10^8^, and the normalized Poisson mean *λ*_0_ of untreated input was set to 10^−6^, unless otherwise clarified. To simulate differential expression, reads counts of each gene in both the IP and the input control sample also vary in a certain range compared with the untreated condition, respectively; and we assume its log2 fold change follows a uniform distribution between [−3,3]. To mimic differential methylation, the methylation reads counts log2 odds ratio follows a uniform distribution between [−3,3] for differential methylation bins and 0 for nondifferential bins. In order to impose dependency of adjacent bins on the simulated data, we applied a definite HMM to generate the labels used as the hidden DMS of the 1000 adjacent bins to indicate whether a bin is differential methylated or not. Then the label was used to generate the data and also used as the ground truth for evaluating the performance of the proposed FET-HMM approach. The transition matrix *A*_sim_ was set as

(17) $$\begin{array}{c} A_{\text{sim}}=\left[\begin{array}{cc} 0.9 & 0.1\\ 0.1 & 0.9 \end{array}\right] \end{array}$$

unless otherwise stated, and the initial probability *π* = (0.5,0.5) due to the lack of prior information. We considered three factors that may affect the performance of the algorithm, that is, the cut-off threshold applied to FET result for switching FDR (or *p* values) to the binary observed state (only for FHB), the transition matrix (degree of spatial dependency) used to generate the ground truth, and the sequencing depth (library size) of the data. The area under receiver operating characteristics curve (AUC) is calculated to evaluate the performance of the proposed algorithms under different settings of the 3 key factors to be tested.

**Figure 5:**
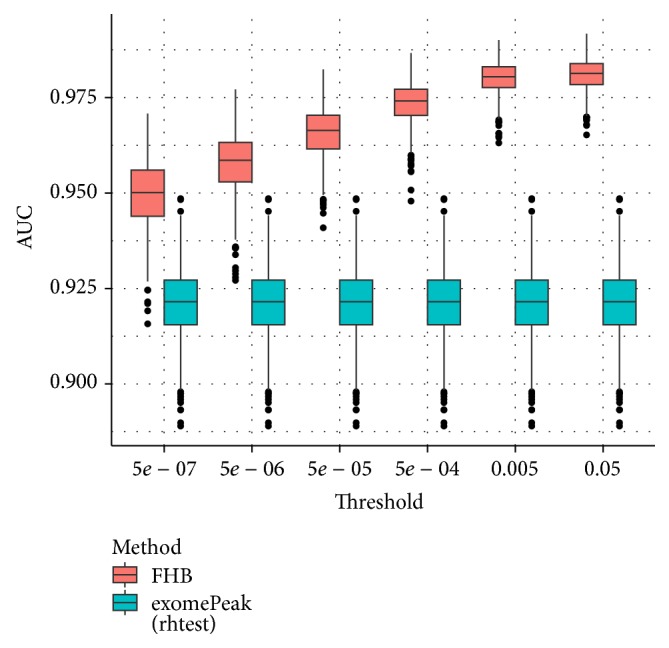
Boxplot of AUCs for different thresholds applied to switch FDR to the binary state. This figure shows that with the variation of thresholds, the performance of FHB outperforms exomePeak in AUC on 100 datasets. exomePeak does not use the cut-off threshold so its performance remains the same. The performance is evaluated at bin level rather than peak level in all experiments.

In the first experiment, we tested the impact of cut-off threshold on the FHB strategy. As shown in Figure 5, although the choice of threshold does affect the performance of the algorithm, by incorporating spatial dependency, the proposed FHB strategy effectively improves the DMRs detection performance under all cut-off thresholds tested.

**Figure 6:**
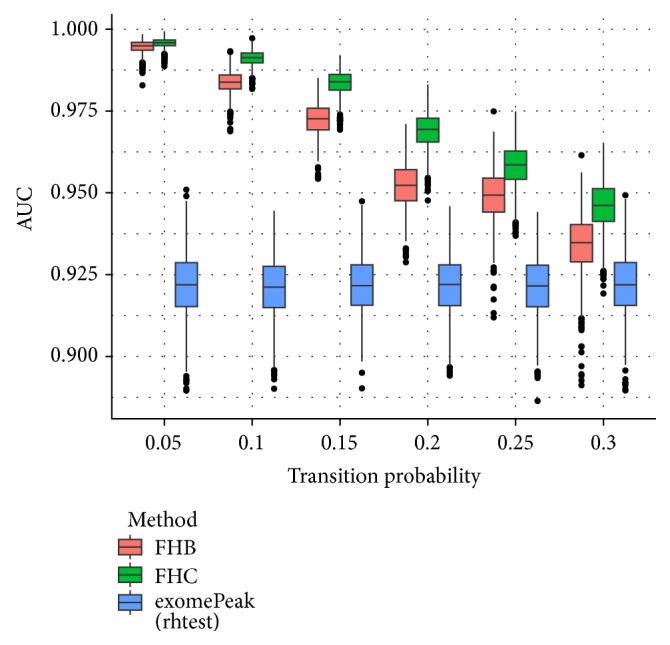
Boxplot of AUCs for different transition matrices used to generate the ground truth. The performance of FHB and FHC strategies heavily relies on the transition matrix setting, which reflects the degree of dependence between adjacent bins; and FHC strategy outperforms FHB and exomePeak under different settings tested.

In the second experiment, we tested the impact of transition matrix, which indicates the degree of dependency between adjacent observations (bins). As shown in Figure 6, the performance of FHB and FHC strategies heavily relies on the transition matrix setting, which reflects the degree of dependence between adjacent bins; and FHC strategy outperforms FHB and exomePeak under different settings tested.

**Figure 7:**
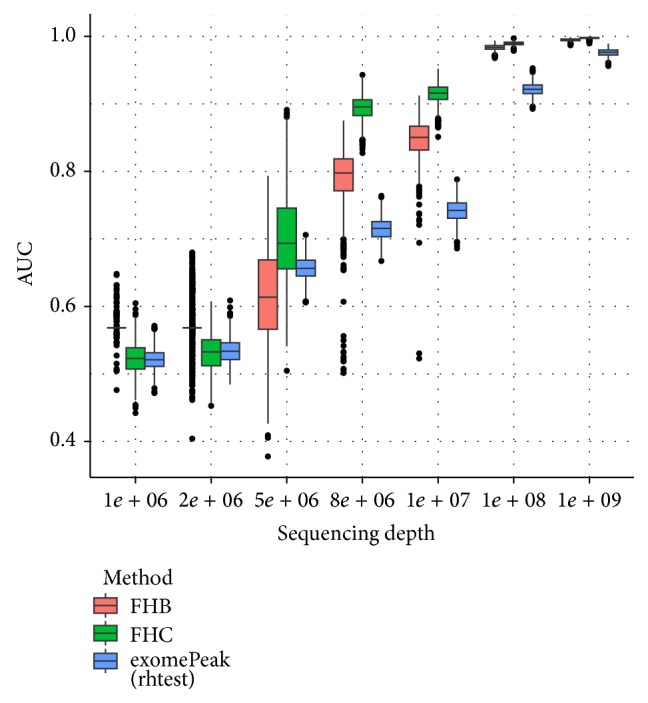
Boxplot of AUCs for different sequencing depths. The performance of all 3 approaches decreases together with the sequencing depth. FHC strategy gives the best performance and the advantage of FET-HMM over exomePeak is the most prominent when the data is of mediocre sequencing depth.

The last factor that may affect the simulation results is the sequencing depth (the total number of reads). In our simulation, the sequencing depths (SD) of the four samples varied from 10^9^ to 10^6^. From Figure 7, we can see that the performances of FHB, FHC, and exomePeak are all satisfactory when sequencing depth is high enough (SD = 10^9^); their performance all decreases together with the sequencing depth. Among the 3 methods tested, FHC gives the best performance and the advantage of FET-HMM over exomePeak is the most prominent when the sequencing depth is low. When the sequencing depth is very low, none of the 3 approaches can identify DMRs effectively.

**Figure 8:**
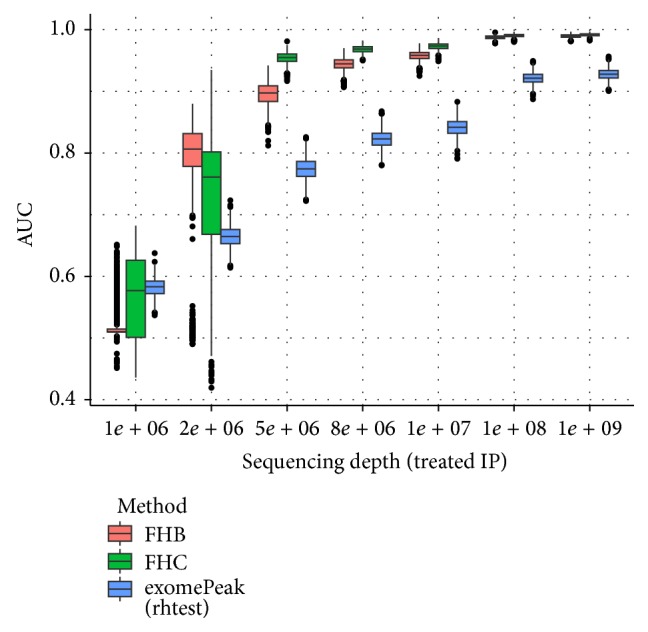
Boxplot of AUCs for different unbalanced sequencing depths. The performance of all 3 approaches decreases as the sequencing depth decreases and FHC strategy outperforms FHB and exomePeak on most settings. In this test, the sequencing depth of IP sample under treated condition varies with that of the other 3 samples unchanged.

We also consider here another scenario of unbalanced sequencing depth; that is, only one of the 4 samples has very large or small sequencing depth, and the results are highly consistent with previous result. As shown in Figure 8, the performance of all 3 approaches decreases as the sequencing depth decreases and FHC strategy outperforms FHB and exomePeak on most settings.

In general, the computational complexity of the proposed approaches increases together with the number of the genes, the length of the genes, and the resolution of the analysis (the size of the bin); and since FHB and FHC require iterative refinement, their computational complexity is also proportional to the number of iterations required to research convergence. To further evaluate the computational complexity of the 3 strategies, we conducted one additional experiment. In this experiment, we simulated a dataset of 7 genes, each with a different length (50, 100, 150, 200, 250, 300, and 350) and the methylation state transition probability is set to be 0.95. A total of 10 datasets are generated for evaluation purposes and the average performance and time consumption are calculated. As it can be seen from Table 1, on the simulated setting, FastFHC is comparable to FHB and FHC in performance, but much faster, making it a reasonable choice for genome-scale data with more than a few thousands of genes.

### 3.2. Test on MeRIP-Seq Data

In order to test our proposed method in real applications, we chose the human MeRIP-Seq data from Hela cells and from METTL3/METTL14 knockout conditions [40] as shown in Table 2. Previous study shows that METTL3 and METTL14 are components of RNA methyltransferase complex [40, 41], and we would like to identify their respective targeted RNA methylation sites from the following analysis. The original raw data in SRA format was downloaded directly from Gene Expression Omnibus (GEO) GSE46705, which consists of 8 IP and 8 Input MeRIP-Seq replicates obtained under wild type condition and after METTL3 or METTL14 knockout, respectively (a total of 16 libraries). The short sequencing reads are firstly aligned to human genome assembly hg19 with Tophat2 [42], and then the same types of samples obtained under the same condition are merged together for differential RNA methylation analysis.

Differential RNA methylation is predicted using exomePeak R/Bioconductor package [21] with UCSC gene annotation database [43] and with FastFHC strategy for comparison. Since METTL3 and METTL14 are methyltransferase, their target sites should exhibit hypomethylation under knockout condition. The hypomethylation sites under knockout condition (targeted RNA methylation sites) are then extracted and their sequences are submitted to MEME-ChIP for motif discovery. The identified motifs are summarized in Table 3. The enriched motifs are quite different in both datasets, indicating that there are multiple regulatory avenues to regulate the RNA methylome through sequence specificity.

**Figure 9:**
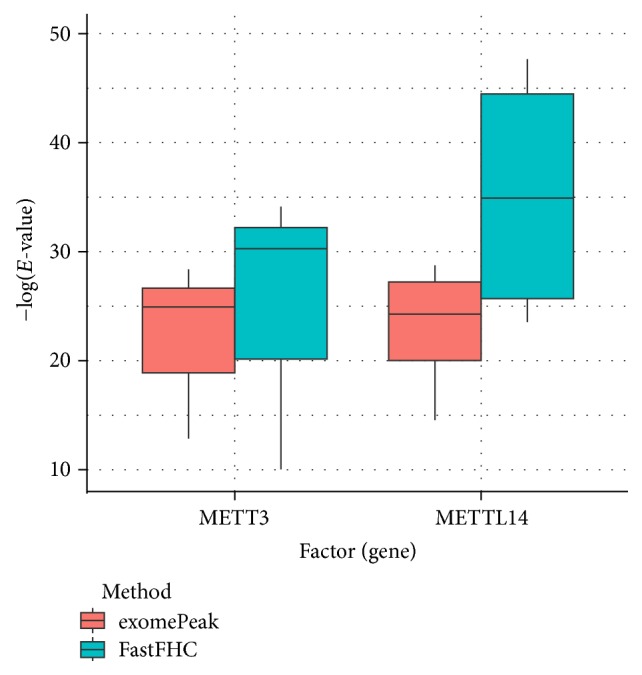
*E* values of motifs identified from differential methylation regions. The figure shows the motif *E* values from exomePeak and FastFHC strategy. With spatially enhanced differential methylation analysis, FastFHC identifies RNA methylation sites that are more biologically meaningful, indicating higher specificity compared with the exomePeak result.

Despite the difference in sequences, as shown in Figure 9, the motifs identified by FastFHC results are more statistically significant than that from exomePeak, indicating higher sequence specificity, which is achieved by spatial enhancement with HMM in FET-HMM approach. The increased sequence specificity will be invaluable for decoding the structure of RNA methylation/demethylation enzymes.

**Figure 10:**
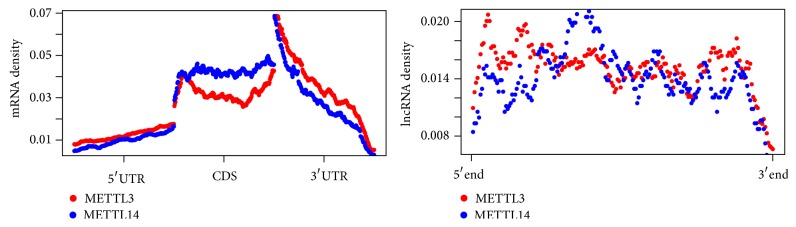
Distribution of METTL3 and METTL14 targeted RNA methylation sites. For both METTL3 and METTL14, their targeted RNA methylation sites are relatively enriched near stop codon of mRNA; however, compared with METTL14 targets, METTL3 targets are relatively enriched on untranslated regions (5′ and 3′UTR). On lncRNA, their targets are unfirmly distributed with slightly enriched on 5′ end.

We then checked the distribution of METTL3 and METTL14 targeted RNA methylation sites on mRNA and lncRNA. As shown in Figure 10, the targeted RNA methylation sites of METTL3 and METTL14 are relatively enriched near stop codon of mRNA. Interestingly, compared with METTL14 targets, METTL3 targets are relatively enriched on untranslated regions (5′ and 3′UTR), which is never reported before. Although existing studies suggest METTL3 and METTL14 function as an RNA methylation complex together with WTAP, our observation suggests that they may have their own respective functions as well. On lncRNA, their targets are almost uniformly distributed on the entire RNA with slight enrichment on 5′ end, whose reason is not yet clear.

## 4. Conclusion

In this paper, we developed an HMM-based method, FET-HMM, for spatially enhanced detection of differentially methylated region from MeRIP-Seq data. Compared with existing peak-based approaches which perform differential analysis on the entire methylation site, FET-HMM seeks to increase the resolution of detection to some extent by dividing the single RNA methylation site into multiple adjacent bins (as shown in Figure 1), resulting in the improved detection performance. We developed 3 different strategies for this purpose, each with different advantage and disadvantages, and the FastFHC strategy can be directly applied to genome scale dataset. We show on the simulated and real datasets that the proposed approaches outperform original approach in detection performance and report more statistically significant DMRs on real MeRIP-Seq data.

It is important to note that exomePeak, which adopts a hypothesis testing scheme, relies on a cut-off threshold to report differential methylation sites, while FET-HMM, which assumes a hidden Markov model, needs a cut-off threshold for posterior probability. Although their performances can be compared under AUC, the two approaches are fundamentally different. It is suggested that both exomePeak and FET-HMM are used when analyzing specific datasets rather than using one approach only.

The proposed approach still has a number of limitations, many of which are shared by other existing MeRIP-Seq data analysis software. Firstly, the proposed approach could not model the within-group variation and thus cannot effectively take advantage of biological replicates. Currently, replicates are merged together which loses the biological variability. Secondly, the proposed approach cannot discriminate different isoforms of the same genes. MeRIP-Seq intrinsically poses very limited information regarding the methylation states of different isoform transcripts. Thirdly, even with the proposed approach, the spatial resolution is still not base-pair resolution. To obtain true base-pair solution, a more advanced computational approach needs to be developed to further combine the nucleotide sequence information (motif).

## Figures and Tables

**Table 1:** Comparison of different approaches.

Method	AUC	Time
FHB	0.960	4.39 s
FHC	0.987	0.85 s
FastFHC	0.962	0.12 s
exomePeak (rhtest)	0.924	0.02 s

**Table 2:** MeRIP-Seq data used.

Dataset	Cell	Treatment	Replicates (IP/input)	Reference
1	Hela	Control	4 & 4	[40]
2	Hela	METTL3 K/O	2 & 2	[40]
3	Hela	METTL14 K/O	2 & 2	[40]

**Table 3:** Motifs for target sites of METTL3 and METTL14.

	Rank	exomePeak		FET-HMM	
		Motif	*E*-value	Motif	*E*-value
	1	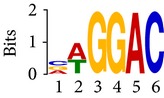	2.3 × 10^−27^	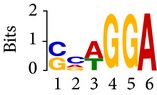	2.4 × 10^−33^
METTL3 K/O	2	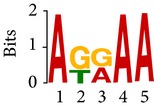	4.7 × 10^−13^	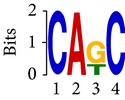	7.1 × 10^−24^
	3	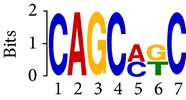	1.5 × 10^−11^	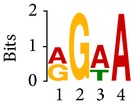	1.5 × 10^−15^
	4	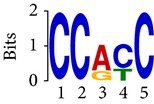	2.6 × 10^−6^	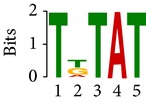	4.4 × 10^−5^

	1	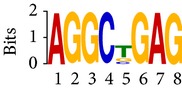	3.3 × 10^−13^	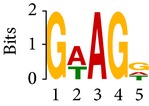	1.4 × 10^−19^
METTL14 K/O	2	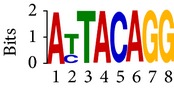	2.5 × 10^−12^	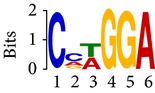	2.0 × 10^−21^
	3	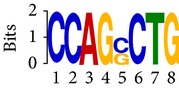	3.3 × 10^−10^	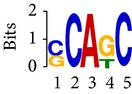	3.4 × 10^−12^
	4	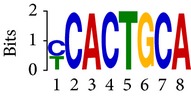	4.8 × 10^−7^	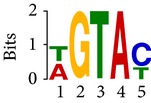	6.0 × 10^−11^
